# Effect of Perioperative Palliative Care on Health-Related Quality of Life Among Patients Undergoing Surgery for Cancer: A Randomized Clinical Trial

**DOI:** 10.1001/jamanetworkopen.2023.14660

**Published:** 2023-05-31

**Authors:** Rebecca A. Aslakson, Elizabeth Rickerson, Bridget Fahy, Brittany Waterman, Rachel Siden, Kathryn Colborn, Shelby Smith, Mae Verano, Isaac Lira, Caroline Hollahan, Amn Siddiqi, Kemba Johnson, Shivani Chandrashekaran, Elizabeth Harris, Richard Nudotor, Joshua Baker, Shireen N. Heidari, George Poultsides, Alison M. Conca-Cheng, Allyson Cook Chapman, Anna Sophia Lessios, Laura M. Holdsworth, Jillian Gustin, Aslam Ejaz, Timothy Pawlik, Judi Miller, Arden M. Morris, James A. Tulsky, Karl Lorenz, Jennifer S. Temel, Thomas J. Smith, Fabian Johnston

**Affiliations:** 1 Department of Anesthesiology, Lerner College of Medicine at the University of Vermont, Burlington; 2 Department of Psychosocial Oncology and Palliative Care, Dana-Farber Cancer Institute, Boston, Massachusetts; 3 Department of Anesthesiology, Perioperative and Pain Medicine, Brigham and Women’s Hospital, Boston, Massachusetts; 4 Department of Surgery, Divisions of Surgical Oncology and Palliative Medicine, University of New Mexico, Albuquerque; 5 Department of Internal Medicine, Division of Palliative Medicine, Ohio State University Wexner Medical Center, Columbus; 6 Division of Primary Care and Population Health, Department of Medicine, Stanford School of Medicine, Stanford, California; 7 Department of Surgery, University of Colorado Anschutz Medical Campus, Aurora; 8 Department of Biostatistics and Informatics, Colorado School of Public Health, Aurora; 9 Clinical Research Department, University of New Mexico Comprehensive Cancer Center, Albuquerque; 10 Department of Surgery, Johns Hopkins Medical Institutions Campus, Baltimore, Maryland; 11 Clinical Research Center, Ohio State University Wexner Medical Center, Columbus; 12 Duke University School of Medicine, Durham, North Carolina; 13 Harvard Medical School, Boston, Massachusetts; 14 Veterans Affairs Boston Healthcare System, Boston, Massachusetts; 15 Department of Medicine, Stanford University School of Medicine, Stanford, California; 16 Department of Surgery, Stanford University School of Medicine, Stanford, California; 17 Department of Pediatrics, Duke Children’s Hospital, Durham, North Carolina; 18 Departments of Medicine and Surgery, University of California, San Francisco; 19 Department of Surgery, Division of Surgical Oncology, Ohio State University Wexner Medical Center, Columbus; 20 Patient Family Advocate, Baltimore, Maryland; 21 Division of Palliative Medicine, Department of Medicine, Brigham & Women’s Hospital, Boston, Massachusetts; 22 VA Palo Alto Healthcare System, Palo Alto, California; 23 Department of Medicine, Division of Hematology/Oncology, MGH, Boston, Massachusetts; 24 Departments of Medicine and Oncology, Johns Hopkins Medical Institutions Campus, Baltimore, Maryland

## Abstract

**Importance:**

Involvement of palliative care specialists in the care of medical oncology patients has been repeatedly observed to improve patient-reported outcomes, but there is no analogous research in surgical oncology populations.

**Objective:**

To determine whether surgeon–palliative care team comanagement, compared with surgeon team alone management, improves patient-reported perioperative outcomes among patients pursuing curative-intent surgery for high morbidity and mortality upper gastrointestinal (GI) cancers.

**Design, Setting, and Participants:**

From October 20, 2018, to March 31, 2022, a patient-randomized clinical trial was conducted with patients and clinicians nonblinded but the analysis team blinded to allocation. The trial was conducted in 5 geographically diverse academic medical centers in the US. Individuals pursuing curative-intent surgery for an upper GI cancer who had received no previous specialist palliative care were eligible. Surgeons were encouraged to offer participation to all eligible patients.

**Intervention:**

Surgeon–palliative care comanagement patients met with palliative care either in person or via telephone before surgery, 1 week after surgery, and 1, 2, and 3 months after surgery. For patients in the surgeon-alone group, surgeons were encouraged to follow National Comprehensive Cancer Network–recommended triggers for palliative care consultation.

**Main Outcomes and Measures:**

The primary outcome of the trial was patient-reported health-related quality of life at 3 months following the operation. Secondary outcomes were patient-reported mental and physical distress. Intention-to-treat analysis was performed.

**Results:**

In total, 359 patients (175 [48.7%] men; mean [SD] age, 64.6 [10.7] years) were randomized to surgeon-alone (n = 177) or surgeon–palliative care comanagement (n = 182), with most patients (206 [57.4%]) undergoing pancreatic cancer surgery. No adverse events were associated with the intervention, and 11% of patients in the surgeon-alone and 90% in the surgeon–palliative care comanagement groups received palliative care consultation. There was no significant difference between study arms in outcomes at 3 months following the operation in patient-reported health-related quality of life (mean [SD], 138.54 [28.28] vs 136.90 [28.96]; *P* = .62), mental health (mean [SD], −0.07 [0.87] vs −0.07 [0.84]; *P* = .98), or overall number of deaths (6 [3.7%] vs 7 [4.1%]; *P* > .99).

**Conclusions and Relevance:**

To date, this is the first multisite randomized clinical trial to evaluate perioperative palliative care and the earliest integration of palliative care into cancer care. Unlike in medical oncology practice, the data from this trial do not suggest palliative care–associated improvements in patient-reported outcomes among patients pursuing curative-intent surgeries for upper GI cancers.

**Trial Registration:**

ClinicalTrials.gov Identifier: NCT03611309

## Key Points

**Question:**

Does proactive specialist palliative care improve patient-reported outcomes in patients receiving curative-intent surgeries for high morbidity and mortality upper gastrointestinal cancers?

**Findings:**

In this randomized clinical trial across 5 geographically diverse cancer centers that included 356 adults randomized to either surgeon alone or surgeon–palliative care team comanagement, there was no significant difference in postoperative patient-reported outcomes, such as quality of life or mental health. Palliative care was well tolerated by study patient and practitioner participants with no associated harms.

**Meaning:**

Data from this study do not suggest benefits with palliative care for surgical oncology patients with newly diagnosed cancers who were pursuing curative-intent operations; integration of palliative care specialists could possibly be more effectively targeted toward patients with poorer baseline quality of life and functionality.

## Teaser

This randomized clinical trial evaluates the use of surgeon–palliative care team comanagement to improve health-related quality of life among individuals undergoing curative-intent surgery for upper gastrointestinal cancers.

## Introduction

Although major surgery is well documented to be safe, perioperative morbidity and mortality are not inconsequential and patients can develop psychological and physical symptoms.^1,2,3,4,5,6,7^ Upper gastrointestinal (GI) cancers typically require extensive surgeries with increased cancer-related mortality and persistent pain, decreased quality of life, poorer global health, diminished appetite, and decreased emotional and social functioning for months to years.^8,9^ Patients pursuing pancreaticoduodenectomy surgery for pancreatic adenocarcinoma have a median survival of 18 months, a greater than 85% 5-year mortality, and postoperative morbidities including delayed gastric emptying, pancreatic fistulae, bilomas, and biliary strictures.^8,10,11^ Esophagectomy surgery may have a perioperative mortality rate of less than 5% but moderate morbidity ranges from 40% to 60%, and 24% of patients experience major morbidity including postoperative bleeding, anastomotic leak, pneumonia, or prolonged postoperative intubation.^3,7^ Curative-intent surgical resections for upper GI cancers are extensive and frequently require postoperative admission to an intensive care unit. Palliative care is patient-centered care that symptomatically and psychosocially supports patients with serious illness and optimizes quality of life, regardless of diagnosis, prognosis, or care goals.^12^ Multiple studies among medical oncology patients support that palliative care improves diverse patient-reported outcomes,^13,14,15,16,17,18^ but few to no studies have evaluated proactive palliative care among a surgical oncologic patient population.^19^ Indeed, multiple studies document surgical culture resistance to palliative and end-of-life care.^20,21,22,23,24,25,26,27,28,29^

Through past research work, some members of our research team identified that patients with upper GI cancers pursuing curative-intent surgeries are a population likely to benefit from proactive palliative care.^30,31,32,33,34,35^ In this population, our trial goal was to evaluate the effect of palliative care integrated with standard surgical oncology care on patient-reported outcomes. We hypothesized that, analogous to studies in medical oncology, proactive palliative care among surgical oncology patients would be associated with better quality of life and mood symptoms.

## Methods

### Study Design

From October 20, 2018, to March 31, 2022, we enrolled ambulatory patients with newly diagnosed upper GI cancers in a nonblinded pragmatic randomized clinical trial to receive either surgeon-alone management (enhanced usual care) or surgeon–palliative care comanagement (intervention). The study was performed at 5 geographically diverse sites including The Johns Hopkins Hospital in Baltimore, Maryland; the Dana-Farber Cancer Institute in Boston, Massachusetts; Stanford University Medical Center in Stanford, California; the University of New Mexico Medical Center in Albuquerque; and The Ohio State University Wexner Medical Center in Columbus. Eligible patients were enrolled when scheduled for surgery and computer randomized in a 1:1 ratio in blocks of 4 and stratified by enrollment site with allocation provided at the time of randomization. Patients assigned to the intervention met either in person or via telephone with a member of an interprofessional palliative care specialist team, which consisted of board-certified palliative care physicians, advanced-practice nurses, social workers, pharmacists, and/or chaplains. Palliative care visits occurred before surgery, 1 week after surgery, and 1, 2, and 3 months after surgery, with additional visits scheduled at the discretion of the patient, surgery team, or palliative care team. The protocol (Supplement 1) was approved by the institutional review board at each institution and all participants provided verbal informed and written consent was later requested at some study sites. Participation in the trial was not compensated but participants would receive a $20 gift card for completion of services (maximum, $100). The full study protocol was previously published.^36^ The study followed the Consolidated Standards of Reporting Trials (CONSORT) reporting guideline for randomized studies.

Guidelines for palliative care visits (Table 1) were adapted from the National Consensus Project for Quality Palliative Care^37^ based on interventions used in previous palliative care clinical trials among medical oncology patients^13,14^ and included in the study protocol.^36^ Palliative care clinicians received training materials about upper GI cancers and operations prepared by a study member board certified in both surgery and palliative care and with content approved by key study team surgeons. Intervention group patients also received routine surgical oncologic care. As consistent with standard practice and adherent to an intention-to-treat analysis, patients in the surgeon-only group could receive palliative care consultation if requested by their surgical team. The surgeon-alone group was considered enhanced usual care as participating surgeons were encouraged to follow the National Comprehensive Cancer Network–recommended triggers for when to involve palliative care consultants^38^ although, as consistent with standard practice, the surgical team decided whether or when to consult palliative care consultants. To facilitate intervention implementation and fidelity, site leadership teams met weekly to discuss study events and one of us (R.A.A.) periodically checked for intervention fidelity through review of deidentified palliative care notes from patients in the intervention arm.

**Table 1.** Components of Surgeon–Palliative Care Team Comanagement

Team element	Description
Time	A goal of at least 60 min/mo (per patient and caregiver preference) devoted to palliative care treatment
Education	Patients and family members, per their wishes, counseled and educated about the disease, including self-management of symptoms, prognosis, and treatment options
Assessment	Formal assessment of symptoms, including pain, dyspnea, constipation/diarrhea, anxiety/depression, fatigue, and nausea
Multidisciplinary	Access to a multidisciplinary palliative care team composed of nurse, physician, social worker, pharmacist, and/or chaplain team members

### Patients

Patients who presented to outpatient hepatobiliary surgery clinics were invited by their surgical oncologist to enroll. Surgeons were encouraged but not required to offer participation to all eligible patients; no additional screening or recruitment measures were used. If a patient declined to participate, study coordinators recorded the reason. Patients were eligible if they were being scheduled for a curative-intent operation for an upper GI cancer and had not previously received specialist palliative care. Patients who did not speak English participated in study activities using a translator service and study materials and activities were available in both English and Spanish.

### Patient-Reported Measures

Health-related quality of life (HRQOL) was measured with the Functional Assessment of Chronic Illness Therapy–Palliative Care (FACIT-Pal) Subscale (46-item metric with scores ranging from 0 to 184; higher scores indicate higher HRQOL),^39^ which includes all elements of the Functional Assessment of Cancer Therapy–General (FACT-G) metric (27-item metric with scores ranging from 0 to 108; higher scores indicate higher HRQOL),^40,41^ which has been used extensively as a quality-of-life outcome in previous medical oncology studies. Patient-Reported Outcomes Measurement Information System (PROMIS) profile summary mental health and physical health scores were calculated from the 29-item (PROMIS-29) tool.^42^ The PROMIS-Preference (PROPr) summary score was developed as a societal-preference–based health-related quality-of-life instrument and is a summary measure of 7 PROMIS-29 domains: cognition, depression, fatigue, pain, physical, sleep, and social; PROPr scores were calculated.^43^

### Data Collection

Participants completed baseline questionnaires at the time of randomization with follow-up questionnaires at 1 week, 1 month, 3 months, and 6 months following the operation. Questionnaires could be completed electronically, on paper, or verbally and could be completed within 2 weeks of the specified date; participants were contacted by email or telephone a maximum of 3 times for survey completion. After each visit, palliative care clinicians completed a survey documenting visit length and content (eAppendix in Supplement 2). Adverse events were patient-, family-, or clinician-reported detrimental events deemed due to involvement of the palliative care specialists; potential adverse events were discussed at weekly site meetings.

### Statistical Analysis

Analysis team members and the study principal investigator (R.A.A.) were blinded to allocation and the analytic data set included data obtained through September 14, 2022. The primary study outcome was group differences in patient-reported HRQOL at 3 months after the operation. Estimated sample size was based on an unpaired, 2-sample *t* test to detect a small to moderate effect size of 0.4 with 90% power and a probability of type I error of .05 (2-sided). We incorporated a variance inflation rate of 20% to account for potential within-site association and assumed a completion rate of 0.86; this attrition rate was based on previously published perioperative mortality rates and past study team experience. Under these assumptions, the estimated sample size was 186 patients per arm for a total of 372 patients. We planned exploratory subgroup analyses among race and ethnicity, and study site subgroups. Hypothesizing a potential association between study outcomes and patient race and ethnicity and/or study site, we planned exploratory subgroup analyses among race and ethnicity and study site groups.

We conducted statistical analyses using R, version 4.2.2 software (R Foundation for Statistical Computing) and used descriptive statistics to estimate the frequencies and means (SDs) of the study variables stratified by study group. We imputed missing outcomes data at 3 months with the nearest postoperative survey. We used *t* tests to compare numeric patient and outcomes variables and χ^2^ tests of association to compare categorical variables between treatment groups. We conducted 2 sensitivity analyses to compare the subset of patients who completed a survey at 3 months postoperatively (no imputation of outcomes) using *t* tests and used linear mixed models to compare the mean treatment effect across all time points for the primary outcome. These models included a binary indicator of treatment group and a random effect to account for within-patient variation across time. To score the PROMIS mental and physical profile scores, we followed the code provided by Spritzer and Hays^44^ to estimate *z* scores.^44^ To score the PROMIS PROPr scores, we applied the R function propr.maut.function.201709 of Hanmer and Dewitt.^45^ Side-by-side boxplots of patient-reported outcome scores were compared by treatment group at each survey time point.

## Results

### Baseline Characteristics of the Patients

**Figure 1.**
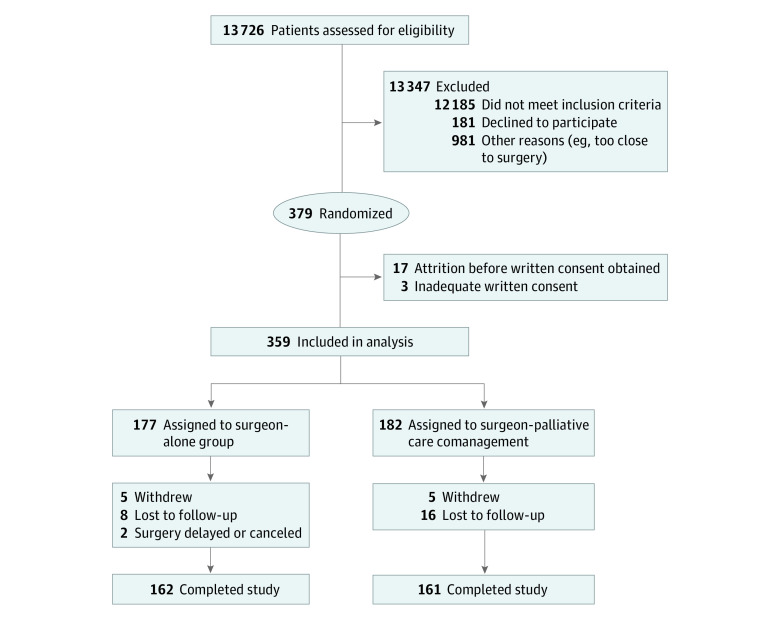
Patient Flowchart

A total of 379 patients were enrolled in the study and verbally consented (Figure 1); 359 patients (175 [48.7%] men; mean [SD] age, 64.6 [10.7] years) were randomized, including 177 participants in the enhanced control arm (mean [SD] age, 64.7 [10.4] years; 90 women [51.4%] and 85 men [51.4%]). Self-reported race and ethnicity categories included American Indian, 1.1%; Asian, 5.1%; Black or African American, 9.1%; Latino or Latina, 10.3%; White or Caucasian, 77.7%; multirace, 0.6%; and other unlisted race, 6.3%. In this cohort, 69.2% individuals had a college degree or higher; cancer types included pancreatic, 61.4%; gastric, 15.2%; esophageal, 6.4%; cholangiocarcinoma, 8.8%; hepatocellular, 4.7%; and other, 3.5%. A total of 182 participants were included in the intervention arm (mean [SD] age, 64.6 [10.9]; 73 women [40.1%]; 109 men [59.9%]). Race and ethnicity categories included American Indian, 2.7%; Asian, 2.2%; Black or African American, 6.0%; Hawaiian or Pacific Islander, 0.5%; Latino or Latina, 12.6%; White or Caucasian, 79.7%; multirace, 1.1%; and other unlisted race, 7.7%. In this cohort, 59.0% of the participants had a college degree or higher; cancer types included pancreatic, 55.8%; gastric, 18.2%; esophageal, 12.2%; cholangiocarcinoma, 7.7%; hepatocellular, 2.2%; and other, 3.9%.

There were no significant changes in methods or collected study outcomes after trial commencement. All patients provided verbal consent, but with changes in written consent practices, particularly surrounding the COVID-19 pandemic, 17 patients could not be contacted for follow-up written consent and 3 had inadequate written consent; data from these 20 patients were excluded. Data from 359 patients were analyzed. Baseline characteristics were similar (Table 2). Consistent with study site variations in surgical volume, enrollment varied by study site. Enrolled patients predominantly underwent operations for 1 of 5 upper GI cancers, with most undergoing curative-intent resection of pancreatic cancer (206 [57.4%] of patients whose malignancy type was known). Loss to follow-up was 8 of 177 patients (4.5%) in the enhanced control arm and 16 of 182 (8.7%) in the intervention arm. A total of 119 eligible patients declined to participate with the most common reasons (patients could select more than 1 reason) being too overwhelmed/stressed by diagnosis/generally tired (n = 64), surgery too soon/don’t want to deal with anything extra (n = 31), and not interested in palliative care/do not see how it would be beneficial/do not want to deviate from current care plan (n = 25).

**Table 2.** Patient Characteristics

Characteristic	No. (%)	
	Enhanced control (n = 177)	Intervention (n = 182)
Study site		
Dana-Farber	47 (26.6)	47 (25.8)
Johns Hopkins	80 (45.2)	83 (45.6)
New Mexico	18 (10.2)	19 (10.4)
Ohio State	3 (1.7)	3 (1.6)
Stanford	29 (16.4)	30 (16.5)
Age, y		
Missing	1	1
Mean (SD)	64.72 (10.40)	64.57 (10.90)
Sex		
Male	85 (48.6)	109 (59.9)
Female	90 (51.4)	73 (40.1)
Missing	2	0
Race		
American Indian or Alaskan Native	2 (1.1)	5 (2.7)
Asian	9 (5.1)	4 (2.2)
Black or African American	16 (9.1)	11 (6.0)
Hawaiian or Pacific Islander	0	1 (0.5)
White or Caucasian	136 (77.7)	145 (79.7)
Multirace	1 (0.6)	2 (1.1)
Other unlisted race	11 (6.3)	14 (7.7)
Missing	2	0
Ethnicity		
Hispanic (Latino or Latina)	18 (10.3)	23 (12.6)
Non-Hispanic	157 (89.7)	159 (87.4)
Missing	2	0
Highest educational level completed		
<8 y	1 (0.7)	2 (1.4)
8-11 y (without graduating high school)	4 (2.8)	4 (2.8)
High school graduation or GED, vocational, or technical school	35 (24.1)	52 (35.9)
College degree	49 (33.8)	53 (36.6)
Graduate, postgraduate, or professional degree	52 (35.9)	31 (21.4)
Prefer not to answer	4 (2.8)	3 (2.1)
Missing	32	37
Marital status		
Never married	13 (7.7)	17 (9.8)
Married	122 (72.2)	120 (69.4)
Divorced	16 (9.5)	18 (10.4)
Domestic partnership/living together	2 (1.2)	4 (2.3)
Separated	1 (0.6)	1 (0.6)
Widowed	14 (8.3)	12 (6.9)
Other	1 (0.6)	1 (0.6)
Missing	8	9
Caregiver		
No	111 (65.7)	117 (67.2)
Yes	58 (34.3)	57 (32.8)
Missing	8	8
Malignancy type		
Pancreatic	105 (61.4)	101 (55.8)
Gastric	26 (15.2)	33 (18.2)
Esophageal	11 (6.4)	22 (12.2)
Cholangiocarcinoma	15 (8.8)	14 (7.7)
Hepatocellular	8 (4.7)	4 (2.2)
Other	6 (3.5)	7 (3.9)
Missing	6	1
Baseline patient HRQOL		
FACT-G		
Missing	30	30
Mean (SD)	81.2 (16.3)	80.7 (17.8)
FACIT-Pal total		
Missing	31	30
Mean (SD)	142.0 (26.1)	140.3 (28.0)
PROMIS-29: physical health		
Missing	44	48
Mean (SD)	−0.05 (0.9)	−0.1 (0.9)
PROMIS-29: mental health		
Missing	44	48
Mean (SD)	0.1 (0.8)	0.1 (0.9)
PROPr PROMIS summary		
Missing	44	48
Mean (SD)	0.5 (0.2)	0.5 (0.2)

Abbreviations: FACIT, Functional Assessment of Chronic Illness Therapy; FACIT-Pal, FACIT–Palliative Care; FACT-G, Functional Assessment of Cancer Therapy–General; GED, general educational development; HRQOL, health-related quality of life; PROMIS-29, Patient-Reported Outcomes Measurement Information System; PROPr, PROMIS-Preference.

### Palliative Care Visits in Both Groups

Among 182 patients randomized to the intervention, 164 individuals (90%) received at least 1 palliative care team visit with a mean (SD) of 3.43 (1.70) of the 5 total possible visits during the first 3 months following the operation; mean visit time was 30 (14.2) minutes (range, 5-75 minutes) and the most common visit content involved relationship/rapport building (71.2%), symptom management (66.7%), illness understanding/education (62.4%), and coping with serious illness (61.3%). No harms associated with the inclusion of palliative care specialists were reported by intervention patients, family members, or clinicians. Among 177 patients randomized to the enhanced control group, 20 (11.3%) had a palliative care consultation during the first 3 months following the operation and 5 were referred for hospice care; 1 study site accounted for 17 of these 20 consultations with 21.3% (17 of 80) of enhanced control arm patients at that site receiving palliative care consultation. Other sites had 4.2% (2 of 47), 3.4% (1 of 29), and 0% (0 of 18 and 0 of 3) of enhanced control arm patients receiving palliative care consultation.

### HRQOL Outcomes

**Figure 2.**
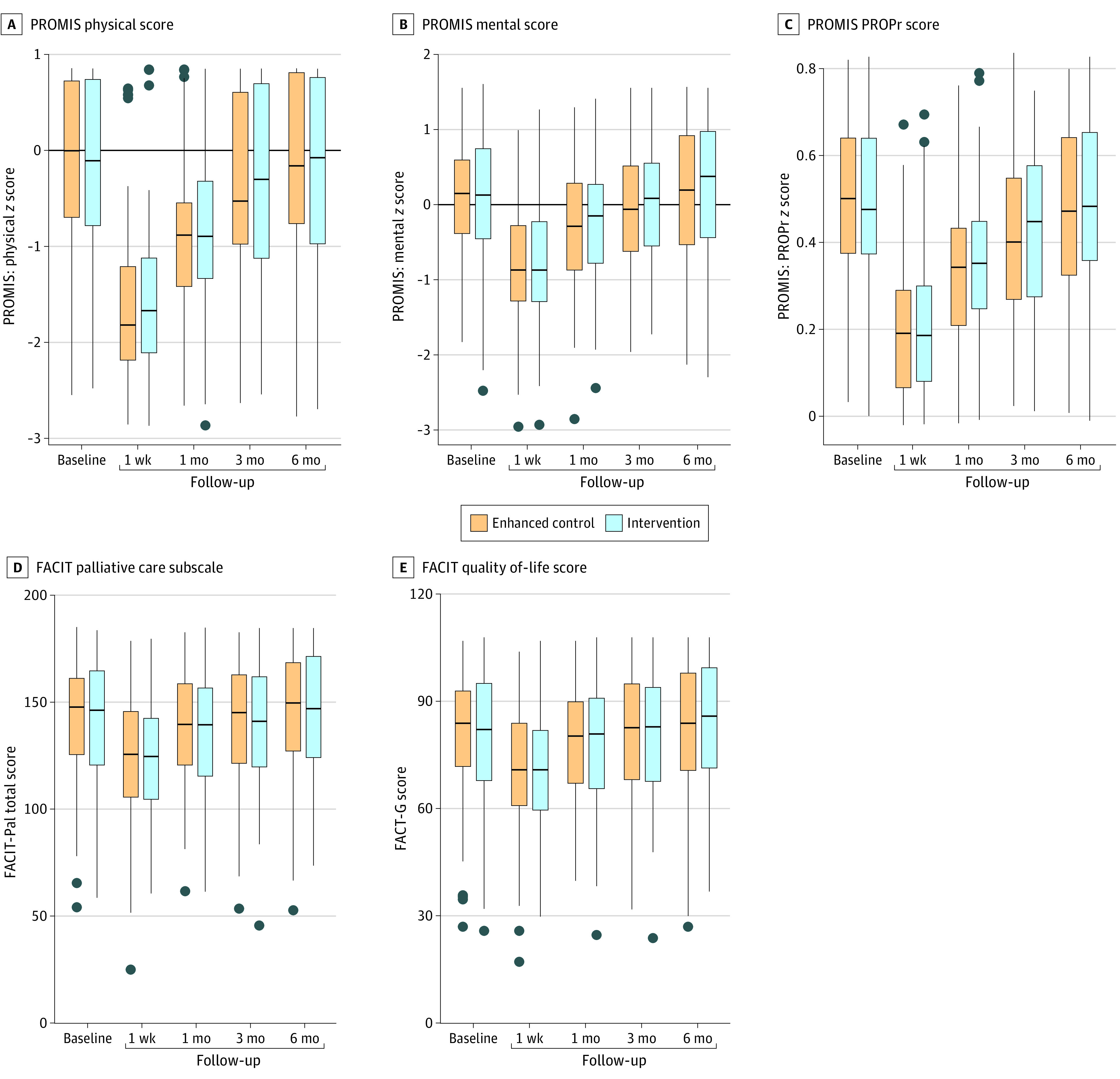
Comparisons of Patient-Reported Outcomes Across Time and Treatment Group Boxes indicate the IQR, black lines within the boxes indicate the medians, lines indicate quartile 1 − 1.5 × IQR (lower) and quartile 3 + 1.5 × IQR (upper). Dots represent potential outliers. FACIT indicates Functional Assessment of Chronic Illness Therapy; FACIT-Pal, FACIT–Palliative Care; FACT-G, Functional Assessment of Cancer Therapy–General; PROMIS, Patient-Reported Outcomes Measurement Information System; PROPr, PROMIS-Preference.

A comparison of measures of HRQOL at 3 months following the operation did not show any significant differences between study groups (mean [SD], FACIT-Pal, 138.54 [28.28] vs 136.90 [28.96]; *P* = .62; FACT-G, 79.90 [17.14] vs 79.40 [17.45]; *P* = .80; PROMIS-29 physical health, −0.43 [0.89] vs −0.50 [1.01]; *P* = .56; PROMIS-29 mental health, −0.07 [0.87] vs −0.07 [0.84]; *P* = .98; and PROPr PROMIS, 0.40 [0.19] vs 0.40 [0.20]; *P* = .83) (Table 3; Figure 2). There was also no significant difference in mortality during the study between the groups (deaths from enrollment until 3 months postoperatively: enhanced control, 6 of 177 [3.7%]; intervention, 7 of 182 [4.1%]; *P* > .99).

**Table 3.** Patient-Reported Outcomes at 3 Months After Surgery

Outcome	Enhanced control (n = 177)	Intervention (n = 182)	*P* value
FACT-G			
Missing, No.	30	30	.80
Mean (SD)	79.90 (17.14)	79.40 (17.45)	
FACIT-Pal total			
Missing, No.	30	30	.62
Mean (SD)	138.54 (28.28)	136.90 (28.96)	
PROMIS-29: physical health			
Missing, No.	32	38	.56
Mean (SD)	−0.43 (0.89)	−0.50 (1.01)	
PROMIS-29: mental health			
Missing, No.	32	38	.98
Mean (SD)	−0.07 (0.87)	−0.07 (0.84)	
PROPr PROMIS summary			
Missing, No.	32	38	.83
Mean (SD)	0.40 (0.19)	0.40 (0.20)	
Died during study, No. (%)			
Alive	156 (96.3)	164 (95.9)	>.99
Died	6 (3.7)	7 (4.1)	
Missing	15	11	

Abbreviations: FACIT-Pal, Functional Assessment of Chronic Illness Therapy–Palliative Care; FACT-G, Functional Assessment of Cancer Therapy–General; PROMIS-29, Patient-Reported Outcomes Measurement Information System; PROPr, PROMIS-Preference.

### Missing Data and Sensitivity Analyses

Of the 128 enhanced control patients who completed a 3-month postoperative survey, 118 completed the FACIT questionnaire and 109 completed PROMIS-29. Among the 141 intervention patients who completed the 3-month postoperative survey, 104 completed the FACIT questionnaire and 87 completed the PROMIS-29. When outcomes were imputed at 3 months, 147 enhanced control patients completed at least 1 patient-reported HRQOL survey and 152 intervention patients completed at least 1 patient-reported HRQOL survey postoperatively. For the sensitivity analysis comparing patient-reported HRQOL at 3 months postoperatively between the 2 arms, among only patients who answered the 3-month survey (ie, no imputed data: enhanced control, 128; intervention, 141), the results were nearly identical with no significant differences between arms. For the sensitivity analysis using linear mixed models, there were again no significant differences between the 2 arms for FACIT-Pal (−1.79; 95% CI, −7.12 to 3.55; *P* = .50), FACT-G (−0.74; 95%, CI, −3.99 to 2.51; *P* = .70), PROMIS physical (0.001; 95% CI, −0.16 to 0.16; *P* > .90), PROMIS mental (0.03; 95% CI, −0.12 to 0.19; *P* = .70), and PROPr (0.001; 95% CI, −0.03 to 0.03; *P* > .90).

## Discussion

To our knowledge, this is the first multisite randomized clinical trial of palliative care in a surgery patient population and includes the earliest reported integration of palliative care in oncologic care. Despite previously discussed surgical cultural barriers to palliative care, this study was completed without any reported palliative care–related harms and was well received by both patients and participating surgeons. Our results do not support that early integration of palliative care among patients with upper GI cancers pursuing curative-intent surgeries improves patient-reported HRQOL or mood symptoms.

Studies support that early integration of palliative care improves patient-reported HRQOL and mood symptoms, particularly among medical oncology patients with metastatic disease with little chance of cure.^46^ Data also support benefits associated with palliative care among medical oncology patients receiving curative-intent treatments,^15,47^ and in observational studies using administrative data sets of surgery patients.^48^ Our data suggest a more nuanced and context-sensitive ecosystem. The surgical oncology clinical milieu may differ from a medical one with more self-limited patient symptoms surrounding the time of surgery. The potentially curative operation may also elevate hope and lessen existential distress. Moreover, while existing data support that palliative care is not harmful, perhaps there is a time when palliative care—particularly provided by specialists—is less likely to be as helpful. It is plausible that patients with newly diagnosed cancer pursuing curative-intent surgery were too early in their cancer trajectory for specialist palliative care to have a measurable benefit. Palliative care also may be less helpful among patients with good functional status, even if they have a serious illness. Compared with our study patients, those in other palliative care clinical trials frequently had metastatic and/or terminal disease at the time of study enrollment and poorer baseline quality-of-life scores. Palliative care delivery in cancer care has also substantially changed over the past decade with increased quality and quantity of palliative care integrated within standard oncologic treatment, even when palliative care specialists may not be explicitly involved. Being less depressed at this early stage of their cancer journey, the patients could have been less likely to have an appreciable improvement in mood symptoms similar to that found in previous palliative care studies.^14^

Existing data support a current, widespread, persistent, and substantial shortage of palliative care specialists.^49,50^ Given this work force limitation, our data suggest that nonspecialist palliative care resources may be sufficient for some populations. Our data also suggest variation in existing palliative care consultation practices for surgical oncology patients and that surgical oncology teams may already be selective in determining when to involve palliative care specialists. Moreover, palliative care, particularly when provided by specialists, may be more influential when delivered closer to patient death. Our limited perioperative study timeline may not yet reveal a palliative care–associated benefit precisely because of relatively few patient deaths during the study period.^51,52^

### Limitations

Study limitations include that patient attrition was higher than anticipated and exacerbated by both direct and downstream consequences of the COVID-19 pandemic, although attrition was similar between the 2 groups. While one could hypothesize pandemic-related impacts to study protocols and procedures, these impacts were likely to be similar across study arms and, even before the pandemic and consistent with previous trials with palliative care delivered by telephone,^13,18,53^ our study always offered a telephonic option for palliative care that was highly used and preferred. While our study incorporated patients from diverse geographic areas, most were White, highly educated, and received care from urban and suburban tertiary or quaternary medical centers; study results may be less generalizable among patients who are more ethnically and racially diverse, less educated, and/or who reside in rural areas. Patients in the intervention arm also overwhelming preferred telephone-based palliative care and, after March 2020 and due to the COVID-19 pandemic, all outpatient palliative care was delivered via telephone; the “dose” of palliative care delivered by telephone could have been lower and less likely to impact outcomes. The study patients also started with high HRQOL compared with patients with cancer in many medical oncology studies, which highlights a relative lack of symptoms that palliative care specialists could potentially ameliorate. In addition, this was a pragmatic trial and, while intervention fidelity was monitored, provision of palliative care in the intervention arm was not protocolized and perhaps could have been stronger in effect if more prescriptively delivered.

## Conclusions

Existing data support that proactive palliative care is safe and likely to confer benefit, particularly among patients who have complex symptoms and/or who are approaching the end of life; our data support that palliative care is feasible and safe in surgical patient populations. Given improved acceptance and delivery of palliative care practices and principles within general medical practice, particularly within cancer care, palliative care provided by nonspecialists may be sufficient for some patients, particularly when a serious illness is at an early stage and symptomatic care is straightforward. Specialist palliative care resources are often limited and can likely be targeted toward patients with a more complex status, such as those with more advanced disease, those closer to the time of death, and/or those with complex physical, psychological, and/or social symptoms that may merit involvement of trained and experienced specialists.

## Notes

Supplement 1.
Trial Protocol

Supplement 2. **eAppendix.** Patient Questionnaire

Supplement 3.
Data Sharing Statement
