# Supplementary material for: Effect of Perioperative Palliative Care on Health-Related Quality of Life Among Patients Undergoing Surgery for Cancer: A Randomized Clinical Trial
Source: JAMA Netw Open. 2023 May 31;6(5):e2314660. doi: 10.1001/jamanetworkopen.2023.14660 (PMC10233417; doi:10.1001/jamanetworkopen.2023.14660)
Supplement: Supplement 1. — Trial Protocol [file jamanetwopen-e2314660-s001.pdf]

TITLE: A Multi-Center Randomized Controlled Trial of Perioperative Palliative Care Surrounding Cancer Surgery for Patients and their Family Members

Coordinating Center
Stanford Cancer Institute
875 Blake Wilbur Drive
Stanford, CA 94305

Protocol Director
Rebecca Aslakson, MD PhD
Stanford University

Co-Investigators
Karl Lorenz, MD MS
Stanford University

Arden Morris, MD
Stanford University

George Poultsides, MD MS
Stanford University

Biostatistician
Kathryn Colborn, PhD, MSPH
University of Colorado Anschutz Medical Campus

Project Manager
Rachel Siden
Stanford University

Version Date (6-21-2022)

TABLE OF CONTENTS

PROTOCOL SYNOPSIS **6**

SCHEMA **7**

LIST OF ABBREVIATIONS AND DEFINITION OF TERMS **8**

1. OBJECTIVES **8**
1.1. PRIMARY OBJECTIVE 8
1.2. SECONDARY OBJECTIVES 8

2. BACKGROUND **8**
2.1 STUDY DISEASE 8
2.2 STUDY AGENT/DEVICE/PROCEDURE 9
2.3 RATIONALE 9
2.4 STUDY DESIGN 9
2.5 CORRELATIVE STUDIES BACKGROUND 10

3. PARTICIPANT SELECTION AND ENROLLMENT PROCEDURES **108**
3.1 INCLUSION CRITERIA 10
3.2 EXCLUSION CRITERIA 10
3.3 INFORMED CONSENT PROCESS 11
3.4 REGISTRATION PROCESS 11
3.5 RANDOMIZATION PROCEDURES 11
3.6 STUDY TIMELINE 12

4. TREATMENT PLAN **13**
4.1 GENERAL CONCOMITANT MEDICATION AND SUPPORTIVE CARE GUIDELINES 13
4.2 CRITERIA FOR REMOVAL FROM STUDY 13
4.3 ALTERNATIVES 13

5. INVESTIGATIONAL AGENT/DEVICE/PROCEDURE INFORMATION **13**
5.1 INVESTIGATIONAL AGENT/DEVICE/PROCEDURE 13
5.2 AVAILABILITY 13
5.3 AGENT ORDERING 13
5.4 AGENT ACCOUNTABILITY 13

6. DOSE MODIFICATIONS **13**

7. ADVERSE EVENTS AND REPORTING PROCEDURES **13**
7.1 POTENTIAL ADVERSE EVENTS 13
7.2 ADVERSE EVENT REPORTING 14

8. CORRELATIVE/SPECIAL STUDIES **14**

9. STUDY CALENDAR **15**

10. MEASUREMENT **15**
10.1 PRIMARY AND SECONDARY OUTCOME MEASURES 15
10.2 SECONDARY OUTCOME 15

11. REGULATORY CONSIDERATIONS **18**

11.1 INSTITUTIONAL REVIEW OF PROTOCOL 18
11.2 DATA AND SAFETY MONITORING PLAN 18
11.3 DATA MANAGEMENT 18
11.4 STUDY DOCUMENTATION 19
11.5 SITE COMMUNICATION 19

12. STATISTICAL CONSIDERATIONS 19
12.1 STATISTICAL DESIGN 19
12.2 INTERIM ANALYSES 20
12.3 DESCRIPTIVE STATISTICS AND EXPLORATORY DATA ANALYSIS 20
12.4 PRIMARY ANALYSIS 20
12.5 SECONDARY ANALYSIS 21
12.6 SAMPLE SIZE 21
12.7 CRITERIA FOR FUTURE STUDIES 22

13. REFERENCES 23

PROTOCOL SYNOPSIS

TITLE	A Multi-Center Randomized Controlled Trial of Perioperative Palliative Care Surrounding Cancer Surgery for Patients and their Family Members
INDICATION	Curative-Intent, Surgery for Upper GI cancer
PRIMARY OBJECTIVE(S)	Patient Quality of Life
SECONDARY OBJECTIVE(S)	Patient: symptom experience, spiritual distress, prognostic awareness, health care utilization, and mortality.<br>Caregiver: quality of life, caregiver burden, spiritual distress, and prognostic awareness.
TREATMENT SUMMARY	Participants randomized to the intervention arm receive five (5) visits with a Palliative Care specialist clinician
SAMPLE SIZE	380 participants

SCHEMA

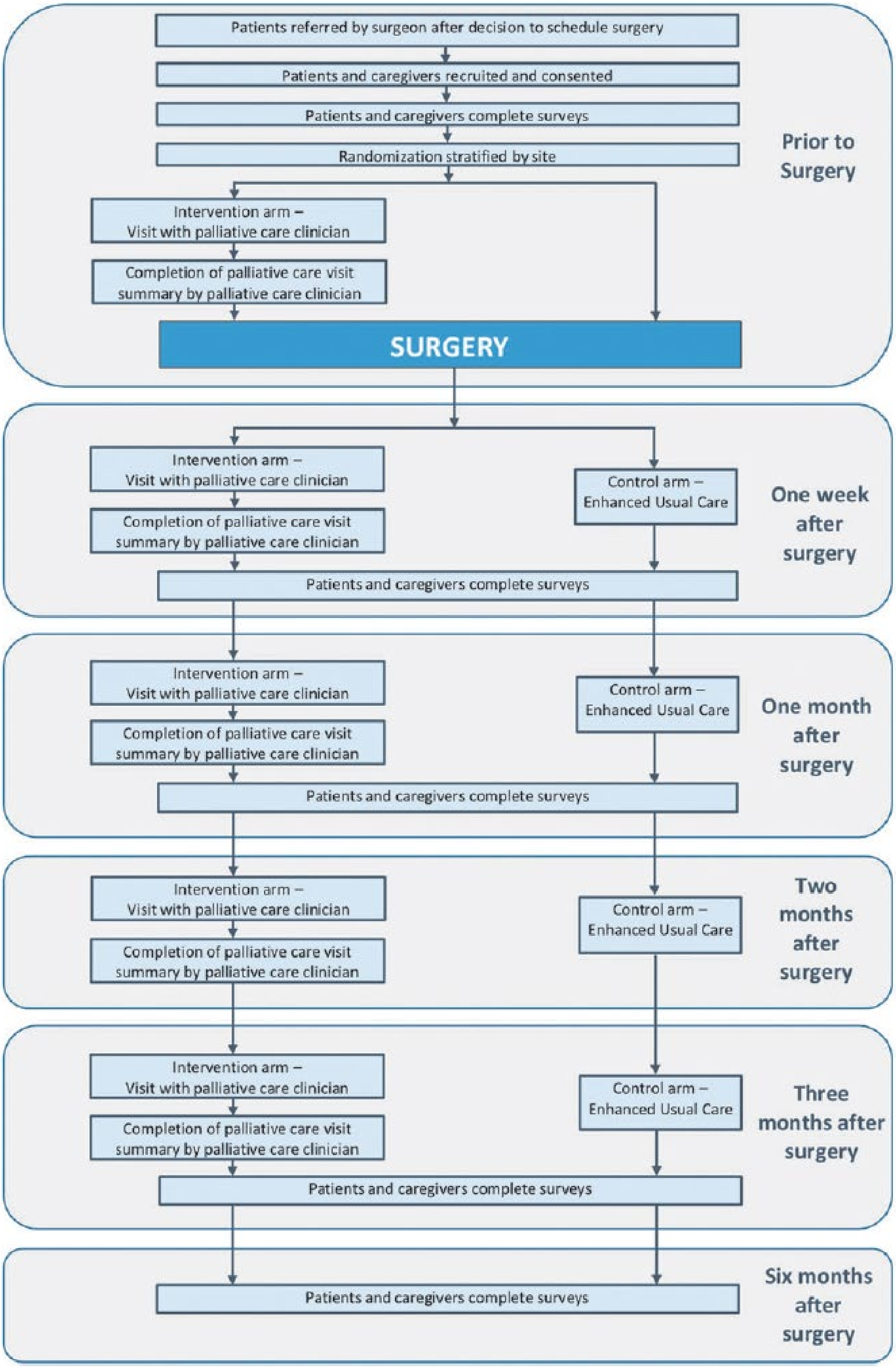

Patients referred by surgeon after decision to schedule surgery
Patients and caregivers recruited and consented
Patients and caregivers complete surveys
Randomization stratified by site
Intervention arm – Visit with palliative care clinician
Completion of palliative care visit summary by palliative care clinician
SURGERY
Prior to Surgery
Intervention arm – Visit with palliative care clinician
Completion of palliative care visit summary by palliative care clinician
Control arm – Enhanced Usual Care
Patients and caregivers complete surveys
One week after surgery
Intervention arm – Visit with palliative care clinician
Completion of palliative care visit summary by palliative care clinician
Control arm – Enhanced Usual Care
Patients and caregivers complete surveys
One month after surgery
Intervention arm – Visit with palliative care clinician
Completion of palliative care visit summary by palliative care clinician
Control arm – Enhanced Usual Care
Two months after surgery
Intervention arm – Visit with palliative care clinician
Completion of palliative care visit summary by palliative care clinician
Control arm – Enhanced Usual Care
Patients and caregivers complete surveys
Three months after surgery
Patients and caregivers complete surveys
Six months after surgery

LIST OF ABBREVIATIONS AND DEFINITION OF TERMS

ADL	Activities of daily living
AE	Adverse event
CRF	Case report/Record form
CR	Complete response
DSMC	Data Safety Monitoring Committee
GI	Gastrointestinal
IRB	Institutional Review Board
PC	Palliative Care
PR	Partial response
PRO	Patient-reported outcomes
RECIST	Response evaluation criteria in solid tumors
RR	Response rate
SAE	Serious adverse event

Of Note: Protocol has been peer-reviewed and published, please see:

RA Aslakson, et. al. "A Multicenter, Randomized Controlled Trial of Perioperative Palliative Care Surrounding Cancer Surgery for Patients and Their Family Members (PERIOP-PC)." Journal of Palliative Medicine.Sep 2019.S-44-S-57.http://doi.org/10.1089/jpm.2019.0130. PMID: 31486730

1. OBJECTIVES

1.1. Primary Objective

Complete a multi-center, comparative effectiveness, randomized controlled trial comparing the impact of surgeon-palliative care team co-management versus surgeon alone management on multiple patient-reported outcomes (PROs), including patient quality of life at 12 weeks after surgery (primary outcome) (n=380; months 1-36).

1.2. Secondary Objectives

Patient: symptom experience, mood symptoms, spiritual distress, prognostic awareness, health care utilization, and mortality. Caregiver: mood symptoms, caregiver burden, spiritual distress, and prognostic awareness.

2. BACKGROUND

2.1 Study Disease

Despite positive outcomes associated with specialist palliative care in diverse medical oncologic populations[1-5], no research has investigated specialist palliative care in surgical oncologic ones[6].

Moreover, although cancer surgery is predominantly safe, operations can be extensive and with not insignificant potential for perioperative morbidity and/or mortality[7-12].

2.2 Study Agent/Device/Procedure

N/A

For clinicaltrials.gov compliance
N/A, this study does not involve any drug or biologic treatment, and does not require an Investigational New Drug application (IND).

2.3 Rationale

Although cancer surgery is safer than ever before, perioperative morbidity and mortality are not inconsequential and studies suggest that cancer patients and their families suffer significant psychological and physical symptoms surrounding surgery and for weeks to months after surgery[7-13]. Palliative care is patient- and family-centered care that symptomatically and psychosocially supports seriously ill patients and their families and optimizes quality of life, regardless of diagnosis, prognosis, or care goals[14,15]. Studies among medical oncology patients support that proactive palliative care: improves quality of life[1-5], betters physical and psychological symptom management[1-5,16], betters understanding of prognosis[17], lessens spiritual distress[4], lessens caregiver burden[4], improves caregiver social well-being[18], decreases caregiver psychological distress[5,18], lowers care costs[16,19,20], decreases aggressive end-of-life care interventions[1,21,22], and may even prolong patient survival[1,22]. Although frequently conflated with hospice[14], *palliative care is for any patient with serious illness and their caregivers*; evidence from a randomized controlled trial in patients undergoing *curative-intent* bone marrow transplantation for treatment of hematologic malignancies supports that proactive palliative care improves patient quality of life, decreases patient physical and psychological symptoms, and decreases caregiver psychological symptoms[5]. Despite these benefits, there have been no studies translating proactive palliative care from a medical oncologic to a surgical oncologic population and none comparing surgeon-palliative care team co-management versus surgeon alone management across patient-reported outcomes (PROs)[6]. Indeed, multiple studies even document surgical culture resistance to palliative care involvement, particularly any discussions that might concern end-of-life care[6,23-26].

2.4 Study Design

For clinicaltrials.gov and Stanford Clinical Trials Directory compliance

This study is a randomized control trial. The primary purpose of this protocol is Supportive Care, designed to evaluate one or more interventions where the primary intent is to maximize comfort, minimize side effects or mitigate against a decline in the subject's health or function. In general, supportive care interventions are not intended to cure a disease. The interventional model is a Parallel study, in which one of two groups in parallel is exposed to the intervention for the duration of the study. There is only one intervention arm. This study is single blinded, in which the PI and analysts are blinded to randomization. The outcomes of this protocol are designed to evaluate Efficacy.

2.5 Correlative Studies Background

N/A, there are no correlative studies planned.

3. PARTICIPANT SELECTION AND ENROLLMENT PROCEDURES

3.1 Inclusion Criteria

3.1.1 Patient is pursuing non-emergent, upper gastrointestinal cancer-related surgery with a goal of primary resection of the tumor – optimal surgical goal is cure, not merely disease palliation.

3.1.2 Eligible cancers for this study include pancreatic, hepatocellular, esophageal, gastric, and/or cholangio-carcinomas.

3.1.3 Must have no previous involvement of specialist palliative care providers in their care course.

3.1.4 Potential study patients must be able to give informed consent and be at least 18 years of age. As assessment for capacity for informed consent is a standard part of the surgical consent process, no patient is referred for the study without having been seen by the surgeon and deemed competent per the surgical team standard protocols.

3.1.5 No ECOG or Karnofsky Performance Status will be utilized.

3.1.6 One caregiver per patient is also asked to participate. In addition to being identified by the patient as being a key caregiver throughout the surgery period, these caregivers must also be able to give informed consent and be at least 18 years of age.

3.1.7 Ability to understand and the willingness to sign a written informed consent document.

3.2 Exclusion Criteria

3.2.1 Previous involvement of palliative care providers in patient's care course.

3.2.2 Not having one of the eligible cancers: Pancreatic, hepatocellular, esophageal, gastric, or cholangio-carcinoma.

3.2.3 Pursuit of emergent surgery for the upper gastrointestinal cancer.

3.2.4 No exclusion requirements due to co-morbid disease or incurrent illness.

3.2.5 Pregnant or nursing patients will not be excluded from the study.

3.2.6 Patients who are cancer survivors or those who are HIV-positive will be not excluded from the study.

3.3 Informed Consent Process

All participants are provided a consent form describing the study with sufficient information for participants to make an informed decision regarding their participation. Participants either sign the IRB approved informed consent prior to participation in any study specific procedure or, through a waiver due to the COVID-19 pandemic, provide verbal consent with waiver of signature. The participant receives a copy of the consent document. The original signed copy of the consent document or documentation of the verbal consent under the COVID-19 pandemic-related waiver is retained by the research team.

3.4 Registration Process

A trained research coordinator obtains consent. The research coordinator is familiar with all aspects of the study. Consent is obtained either in-person at Stanford Hospital or clinics after the research coordinator approaches potential patients about the study or via telephone after the study has been introduced by the patient's surgical oncologist. As much time as necessary is devoted to consent discussion, with ample time for patients or caregivers to ask any questions. If participants are unsure about their participation, they have the opportunity to contact the study team prior to their surgery. To minimize the possibility of coercion, the research coordinator emphasizes that participation is completely voluntary and that patients or caregivers are not required to participate in the study. During the consenting process, the research coordinator questions the patient or caregiver to verify that they understand the purpose and summary of the study as what would be required of them; if participants do not understand English or have a hearing impairment, we use translators in the consent process.

To register the subject, the study site utilizes the central Stanford study REDcap database. The individual enters all subject eligibility and consent information. No subject begins treatment prior to registration and assignment of a subject identification number.

At registration, each study site coordinator assigns eligible subject an identification number. This identification number is used on the shared Stanford REDcap database as the record indicator. The subject's identification number is used on all subject-specific Case Report Forms (CRFs) and serious adverse event (SAE) forms. Participant information is entered into Oncore within 7 days.

3.5 Randomization Procedures

Randomization is immediately after enrollment, stratified by study site, and completed via computer-generated random allocation with a block size of 6 by using the REDcap database. Understandably, neither the patient, caregiver, nor surgeon can be blinded to intervention allocation. However, the principal investigator (PI) and analysis team are blinded to participant randomization and the research team acquiring outcome data, whenever possible, is blinded to participant randomization.

3.6 Study Timeline

Primary Completion:
The study was scheduled to reach primary completion 24 months from the time the study opens to accrual. Initial delay in enrollment at some of the study sites and the ongoing COVID pandemic have led to enrollment delays. Yet, the study team communicates closely with the funder (the Patient-Centered Outcomes Research Institute; PCORI); PCORI is committed to this study and its success and willing to support a longer study duration, if that is needed to enable full study enrollment.

Study Completion:
The study was to reach completion approximately 24 months from the time the study opened for accrual. Yet, as previously mentioned, that accrual has been delayed due to study site delays in starting enrollment and the ongoing COVID-19 pandemic. Yet, the study team and PCORI communicate closely and PCORI is supportive of continuing the trial as needed to ensure meeting enrollment goals. Based on the most recent call with our program officer (June 23, 2021), our current tentative end date is December 31, 2022.

Study start date: March 2019.
Anticipated study completion date: December 2022

4. TREATMENT PLAN

This is a multi-center, comparative effectiveness randomized controlled trial comparing the impact of surgeon-palliative care team co-management versus surgeon alone management on PROs, including quality of life (primary outcome), symptom score, caregiver burden and spiritual distress. The two study arms are below:

1) Surgeon alone management (Enhanced control) – considered “enhanced usual care” this involves surgeon and surgical team management of symptoms, psychosocial support, and prognostic-related communication. The surgeon and surgical team care for the patient and their family both prior to and following surgery. As consistent with standard practice, the surgeon may consult the palliative care team at any time, if desired. Per PCORI’s request, this study arm is “enhanced” in that surgical oncologists are provided and encouraged to consider following National Comprehensive Cancer Network guidelines as to when to consider formal palliative care consultation.

2) Surgeon palliative care team co management (Intervention) – All patients receive the care described in the “Surgeon alone management” arm. In addition to this, palliative care is provided by a specialist team and in a way consistent with the palliative care team co-management evaluated in clinical trials of specialist palliative care team co-management for medical oncologic populations. For patients in this arm, patients and/or family members are seen by the palliative care team: (1) prior to surgery, (2) during their hospitalization for their surgery, and (3) on an at least monthly basis until 12 weeks following surgery. Consistent with previous palliative care interventions, post-operative palliative care interactions following patient discharge from the hospital can be in person at the outpatient clinic, via a telehealth visit, or via telephone, Facetime, or Skype, whichever is preferred by the patient and family.

4.1 General Concomitant Medication and Supportive Care Guidelines

N/A, there is no use of concomitant medications or additional appropriate supportive care medications or treatments. Any palliative care-related medication changes for patients in the intervention arm are determined through discussion between the palliative care specialist and surgical teams.

4.2 Criteria for Removal from Study

Participants could be removed from study at any time if they choose to withdraw, or if the surgeon chooses to withdraw the patient from the study.

4.3 Alternatives

N/A, this has been deemed a low-risk study; the only potential risk to participants is considered to be breach of confidentiality.

5. INVESTIGATIONAL AGENT/DEVICE/PROCEDURE INFORMATION

5.1 Investigational Agent/Device/Procedure

N/A

For clinicaltrials.gov and Stanford Clinical Trials Directory compliance

N/A

5.2 Availability

N/A

5.3 Agent Ordering

N/A

5.4 Agent Accountability

N/A

6. DOSE MODIFICATIONS

N/A

7. ADVERSE EVENTS AND REPORTING PROCEDURES

7.1 Potential Adverse Events

We do not anticipate adverse events related to the study, as this is a low-risk complex behavioral study. No investigational agents or procedures are being studied.

7.2 Adverse Event Reporting

Adverse Events will be clearly noted in source documentation and listed on study specific Case Report Forms (CRFs). The Protocol Director (PD) or designee will assess each Adverse Event (AE) to determine whether it is unexpected according to the Informed Consent, Protocol Document, or Investigator's Brochure, and related to the investigation.

Regulatory and reporting requirements

It is the responsibility of the investigator to document all adverse events which occur during the investigation. Anticipated day-to-day fluctuations of the disease under study that do not represent a clinically significant exacerbation or worsening need not be considered an adverse event.

Reporting Serious Adverse Events

N/A there are no investigational agents being studied.

Any SAEs which occur as a result of protocol specific diagnostic procedures or interventions must be reported to the local DSMC and local IRB (using the formed attached or an allowable local form) and to the coordinating site (Stanford University).

8. CORRELATIVE/SPECIAL STUDIES

N/A There are no correlative studies planned in relation to this randomized clinical trial.

9. STUDY CALENDAR

	Pre-Study	1 Week Before Surgery	1 Week After Surgery	1 Month After Surgery	2 Months After Surgery	3 Months After Surgery	4 Months After Surgery	5 Months After Surgery	6 Months After Surgery	Off Study[d]
Palliative Care Visit		X	X	X	X	X				
Informed consent	X									
Demographics	X									
Medical Abstraction										X
FACIT-PaL		X	X	X		X			X	
ESAS		X	X	X		X			X	
FACIT-Sp-12		X	X	X		X			X	
PROMIS-29		X	X	X		X			X	
Presence of Advance Care Planning	X								X	
(Caregivers) ZBI-12, PROMIS 29, FACIT-Sp-12		X	X	X		X			X	
Adverse event evaluation		X…………………………………………………………..……………………..X								X
Other tests, as appropriate										
Other correlative studies										

10. MEASUREMENTS

For clinicaltrials.gov and Stanford Clinical Trials Directory compliance

Note: Each outcome measure listed within the protocol will necessitate legally required results reporting to clinicaltrials.gov within one year after the completion of the primary outcome measure.

10.1 Primary and Secondary Outcome measures

The primary outcome variable of this project is patient-reported quality of life.

10.1.1 Relevant Subset

All participants will be assessed for primary outcome.

10.1.2 Measurement Definition

The primary outcome variable of this project is patient quality of life, measured by the Functional Assessment of Chronic Illness Therapy—Palliative Care (FACIT-PaL) Subscale.

	Study design	Participants	Outcomes and Measurement Times
SA1 (months 1-36)	Randomized controlled trial (n=380 patients;<br>n = 304 family members);<br>Outcomes measured at approximately:<br>- enrollment<br>- 1 week after surgery<br>- 1 month after surgery<br>- 3 months after surgery<br>- 6 months after surgery	Patients	**(1) Quality of life - Functional Assessment of Chronic Illness – Palliative Care (FACIT-Pal)****<br>Subscales: (i) Functional Assessment of Cancer Therapy (FACT-G)<br>(ii) Trial Outcome Index (TOI)<br>(2) Demographics<br>(3) Edmonton Symptom Assessment Score (ESAS)*<br>(4) Functional Assessment of Chronic Illness Therapy – Spiritual Well-being (FACIT-Sp-12)*<br>(5) Mood symptoms including anxiety and depression subscales – PROMIS-29<br>(6) Health care utilization* (# of post-operative hospitalizations and/or emergency room visits)<br>(7) Presence of advance care planning*<br>(8) Prognostic understanding*<br>(9) Mortality*
		Family members/ Companions	(1) Demographics<br>(2) Zarit Caregiver Burden Scale (ZBI-12)*<br>(3) Mood symptoms including anxiety and depression subscales – PROMIS-29<br>(4) Functional Assessment of Chronic Illness Therapy – Spiritual Well-being (FACIT-Sp-12) family/companion measure*<br>(5) Prognostic understanding*

10.1.3 Measurement Methods

The primary outcome variable of this project is patient quality of life, measured by the Functional Assessment of Chronic Illness Therapy—Palliative Care (FACIT-PaL) Subscale[27-29]. This subscale has not been previously used in a surgical population; however, FACIT-PaL includes all of the elements of Functional Assessment of Cancer Therapy-General (FACT-G), which have been used extensively as a quality-of-life outcome in cancer populations.

10.1.4 Measurement Time Points

The outcome of quality of life will be assessed at baseline during enrollment, one week after surgery, one month after surgery, 3 months after surgery, and 6 months after surgery. Participants will have a window of +/-7 days to complete the surveys for each timepoint before it is considered "missed."

10.1.5 Response Review

N/A

10.2 Secondary Outcome

Patient: symptom experience, spiritual distress, prognostic awareness, health care utilization, and mortality. Caregiver: Quality of life, caregiver burden, spiritual distress, and prognostic awareness.

10.2.1 Relevant Subset

All participants will be assessed for secondary outcomes.

10.2.2 Measurement Definition

Secondary outcomes are

Patient—mood symptoms (PROMIS-29[30,31]), spiritual distress (FACIT—Spiritual Well-being[32]), symptom experience (Edmonton Symptom Assessment Score[33]), prognostic awareness (questions adapted from the CANCORS study[34]), health care utilization (# of post-operative hospitalizations and/or emergency room visits), mortality, and self-described experiences and thoughts about surgeon-PC team co-management.

Caregiver—mood symptoms (PROMIS-29[30,31]), spiritual distress (FACIT—Spiritual Well-being[32]), prognostic awareness (questions adapted from the CANCORS study[34]), caregiver burden (Zarit Caregiver Burden Scale—ZBI-12[35]), and self-described experiences and thoughts about surgeon-PC team co-management.

	Study design	Participants	Outcomes and Measurement Times
SA1 (months 1-36)	Randomized controlled trial (n=380 patients; n = 304 family members); Outcomes measured at approximately:<br>- enrollment<br>- 1 week after surgery<br>- 1 month after surgery<br>- 3 months after surgery<br>- 6 months after surgery	Patients	**(1) Quality of life - Functional Assessment of Chronic Illness – Palliative Care (FACIT-Pal)****<br>Subscales: (i) Functional Assessment of Cancer Therapy (FACT-G)<br>(ii) Trial Outcome Index (TOI)<br>(2) Demographics<br>(3) Edmonton Symptom Assessment Score (ESAS)*<br>(4) Functional Assessment of Chronic Illness Therapy – Spiritual Well-being (FACIT-Sp-12)*<br>(5) Mood symptoms including anxiety and depression subscales – PROMIS-29<br>(6) Health care utilization* (# of post-operative hospitalizations and/or emergency room visits)<br>(7) Presence of advance care planning*<br>(8) Prognostic understanding*<br>(9) Mortality*
		Family members/ Companions	(1) Demographics<br>(2) Zarit Caregiver Burden Scale (ZBI-12)*<br>(3) Mood symptoms including anxiety and depression subscales – PROMIS-29<br>(4) Functional Assessment of Chronic Illness Therapy – Spiritual Well-being (FACIT-Sp-12) family/companion measure*<br>(5) Prognostic understanding*

10.2.3 Measurement Methods

- Patient – mood symptoms (PROMIS-29[30,31]), spiritual distress (FACIT—Spiritual Well-being[32]), symptom experience (Edmonton Symptom Assessment Score[33]), prognostic awareness (questions adapted from the CANCORS study[34]), healthcare utilization, mortality, and self-described experiences and thoughts about surgeon-PC team co-management.

- Caregiver—mood symptoms (PROMIS-29[30,31]), spiritual distress (FACIT—Spiritual Well-being[32]), prognostic awareness (questions adapted from the CANCORS study[34]), caregiver burden (Zarit Caregiver Burden Scale—ZBI-12[35]), and self-described experiences and thoughts about surgeon-PC team co-management.

10.2.4 Measurement Time Points

The secondary outcome will be assessed at baseline during enrollment, one week after surgery, one month after surgery, 3 months after surgery, and 6 months after surgery. Participants will have a window of +/-7 days to complete the surveys for each timepoint before it is considered "missed."

10.2.5 Response Review

N/A

11. MULTISITE REGULATORY CONSIDERATIONS

The Stanford Cancer Institute Data and Safety Monitoring Committee (DSMC) will be the monitoring board for this study, please refer to the Data and Safety Monitoring Committee SOP for more information.

11.1 Monitoring plan

The Stanford Cancer Institute Data and Safety Monitoring Committee (DSMC) will be the monitoring entity for this study. The DSMC will audit study-related activities approximately once per year to determine whether the study has been conducted in accordance with the protocol, local standard operating procedures, FDA regulations, and Good Clinical Practice (GCP). This may include review of the following types of documents participating in the study: regulatory binders, case report forms, eligibility checklists, and source documents. In addition, the DSMC will regularly review serious adverse events and protocol deviations from all sites associated with the research to ensure the protection of human subjects. Results of the DSMC audit will be communicated to the IRB and the appropriate regulatory authorities at the time of continuing review, or in an expedited fashion, as needed.

11.2 Protocol Review and Amendments

The protocol, the proposed informed consent and all forms of participant information related to the study (e.g. advertisements used to recruit participants) will be reviewed and approved by the Stanford IRB and Stanford Cancer Institute Scientific Review Committee (SRC). Any changes made to the protocol will be submitted as a modification and will be approved by the IRB prior to implementation. The Protocol Director will disseminate the protocol amendment to all participating investigators. Investigators will be expected to obtain IRB approval within 90 days for all amendments.

11.3 Data management

The electronic dataset and recordings are stored on an encrypted computer that is password protected with a secure server. All paper copies of the consent form are stored in a locked filing cabinet. During the data collection period, only the study team has access to the Stanford-hosted REDCap database that contains protected health information.

All data will be stored in HIPAA compliant Stanford Medicine Box and/or REDcap. All electronic devices used to store study data, including but not limited to: computers, smartphones, tables, external hard disks USB drives, etc. that any hold identifiable participant data will be password protected, backed, up and encrypted per Stanford policy. All participants will be given a unique identifier number with data storage linked to the UIN and all data stored in a password-protected, secure database.

11.4 Study Documentation

The Protocol Director and participating site investigators must maintain adequate and accurate participant case histories with observations and other data pertinent to the study. Original source documents should be transcribed to Case Report Forms (CRFs) and used to communicate study data to the lead site. Source documents include informed consent forms and electronic medical data.

Participating Center's PIs will be responsible for maintaining the clinical protocol and subjects' study charts, reporting adverse events, assuring that consent is obtained and documented, and reporting the status of the trial in continuing renewals submitted to their IRB and trial monitoring group(s) as per their facility protocol.

11.5 Site Communication

Teleconferences are convened weekly between all sites to discuss participants and study-related matters; calls may also occur more frequently if needed. Teleconferences are coordinated by the Stanford site coordinator and include study PI and all research coordinators. Any issues with patient compliance, database entry, or other items will also be discussed in these calls. Once a month, the call includes the study PI, all research coordinators, and all study site PI's.

12. STATISTICAL CONSIDERATIONS

Since funding in 2017, this study has been in collaboration with the Palliative Care Research Cooperative (PCRC), which is based out of the University of Colorado in Denver. The initial study biostatistician was Suwei Wang of Stanford University but Dr. Wang departed from Stanford. Consequently, through the study team's longitudinal relationship with the PCRC, the current study biostatistician is Kathryn Colborn, PhD, MSPH, Assistant Research Professor at the University of Colorado Anschutz Medical Campus in Denver, CO.

12.1 Statistical Design

Differences in primary and secondary outcomes between intervention and control arms will be evaluated at each individual time point as well as across the intervention period.

12.1.1 Randomization

Randomization occurs immediately after enrollment. Participants are stratified by study site. Using the REDCap database, group assignment (intervention or control) is determined via a computer-generated random allocation with a block size of 6. Patients, caregivers, and surgeons cannot be blinded to group assignment due to practical considerations; however, the principal investigator and analysis team are blinded to participant randomization. The research team collecting outcome data are blinded to participant randomization, whenever possible.

12.2 Interim analyses

No interim analyses will be conducted.

12.3 Descriptive Statistics and Exploratory Data Analysis

Descriptive statistics will be calculated to summarize patients' characteristics and other baseline variables. Comparability of the intervention arm and the control arm will be assessed with regard to preintervention sociodemographic and health status measures derived from the Medical Record Abstraction. Although randomization should account for such differences, a two-sample t-test/Mann-Whitney test will be performed to investigate the differences between intervention and control group for continuous variables and Fisher's exact test or chi-squared test will be used to investigate differences between intervention and control group for binary or categorical variables.

For primary and secondary outcomes, based in the type of the data, summary univariate (descriptive) statistics (e.g., mean, standard deviation, median, interquartile range, maximum, minimum, count, percentage) will be determined for all outcomes stratified by group assignment. Descriptive time trend plots (multiple visits) stratified by group assignment will be presented for outcomes that are measured at multiple visits to allow for the visual comparison of change patterns before and after the intervention.

12.4 Primary Analysis

The primary outcome for this study is quality of life three months following surgery.

12.4.1 Analysis Population

Our study will use an intent-to-treat approach in which all data from study patients in both intervention and control arms are used, regardless of the level of adherence to the study arm.

12.4.2. Analysis Plan

The effect of group assignment (intervention or control) on the quality of life will be tested. Differences in outcomes between two arms at each visit will be tested by the two-sample t-test/Mann-Whitney test or Fisher's exact test/chi-squared test based on the data types of the outcomes. The effect of intervention on the quality of life after accounting for various confounding variable will be determined using a linear mixed model that accounts for within-subject variations due to repeated measures. Sensitivity analyses will assess whether there are differential effects on contingent on patient or study site characteristics on the primary outcomes.

The overall level of statistical significance was set at $p<0.05$. For all models, the research team will identify possible confounding variables for model adjustment, including baseline attributes. Using peer-reviewed literature, the research team has identified confounding variables that models will be adjusted for. These covariates include patient gender, age, race, education, and health status.

Qualitative data related to secondary outcomes will be transcribed, de-identified, and analyzed

based on qualitative description [36,37]. A HIPPA-compatible, professional transcription service will be used for interview transcription. NVivo software will be used for qualitative analyses. A codebook will be determined by a three-person team with a single coder then analyzing the transcripts. Line-by-line, axial, and theoretical consensus coding will be used to organize and summarize findings, which will be validated through triangulation, member checking, and search for disconfirming data.

12.5 Secondary Analysis

Secondary outcomes include physical symptom assessment, mood symptoms (measured through a subscale of PROMIS-29), spiritual distress assessment, mortality, and assessment of caregiver burden by the linear mixed model.

12.5.1 Analysis Population

Our study will use an intent-to-treat approach in which all data from study patients in both intervention and control arms are used, regardless of the level of adherence to the study arm.

12.5.2 Analysis Plan

Depending on the format of the variable, the effect of group assignment (intervention or control) on the secondary outcomes will be tested at each visit with two-sample t-test/Mann-Whitney test or Fisher's exact test/chi-squared test. The effect of intervention on secondary variables after accounting for various confounding variables will be determined using a linear mixed model that accounts for within-subject variations due to repeated measures. For end-point secondary outcomes, Kaplan-Meier method or Cox proportional hazards models will be used.

The overall level of statistical significance was set at $p<0.05$. For all models, the research team will identify possible confounding variables for model adjustment, including baseline attributes. Using peer-reviewed literature, the research team has identified confounding variables that models will be adjusted for. These covariates include patient gender, age, race, education, and health status.

12.6 Sample Size

12.6.1 Accrual estimates

Target enrollment was approximately 30 participants per month with 6-10 per site. As of March 31, 2022 and with 34 months of enrollment, we have enrolled 381 participants. Enrollment slowed due to the COVID-19 pandemic. We have been in close communication with PCORI who were supportive of our continuing enrollment, as safe and feasible, through the pandemic until we met our target enrollment goals. PCORI has been supportive of the extended timeline to enroll patients.

12.6.2 Sample size justification

It is hypothesized that surgeon-palliative care co-management (intervention) perioperative palliative care will improve patient postsurgical quality of life as compared to surgeon-alone management of care (control). Thus, the null hypothesis is that the intervention and control groups will not differ in postsurgical quality of life measures.

The estimated sample size is 186 patients per arm or 372 patients total (380 patients was our goal). The sample size and power of this study was based on the primary outcome quality of life measure, FACIT-Pal. With this sample size, using an unpaired two-sample t-test, the present study is powered to detect an anticipated small-to-moderate effect size of 0.4 at 12 weeks with 90% power and probability type I error of 0.05 (two-sided). This power analysis includes a 86% participant completion rate as well as a variance inflation factor of 20%.

One of the study sites had a study coordinator abruptly quit the position in 2019 and consequently data on 20 participants from that site were compromised. Following the same methodological approach described in the published protocol paper (which anticipated a LTFU rate of 14%), we performed a re-estimate of the required sample size for this study in December 2020 and discovered that it was higher than anticipated (28%) due to the study coordinator turnover and interruptions caused by the pandemic. This was discussed with the funder (PCORI); the study team and PCORI had consequently agreed to target 420 enrollees to ensure sufficient sample size and study power. However, in December 2021, we re-evaluated our LTFU rate and found that it had markedly improved during the most recent year of enrollment and was now the same as was originally anticipated. In communication with PCORI, we returned the enrollment rate to 380.

12.6.3 Effect size justification

The means, standard deviations, and effect sizes were based on previous research [1] supporting that patients receiving medical oncologist-palliative care co-management had better quality of life (measured by Functional Assessment of Cancer Therapy – Lung; FACT-L[38]) than patients receiving medical oncologist alone management. Mean score differences in FACT-L were 98.0 ± 16.7 (n = 74) in the intervention group and 91.5 ± 16.5 (n = 77) in the control group resulting in an effect size of 0.42 (unpaired two-sample t-test). Since the FACIT-Pal scale[27-29] used in this study is a direct corollary of the FACT-L[38] scale, this effect size was used to estimate the sample size for the present study. The sample size and power of this study are based on the quality of life measure, FACIT-Pal. Based on the unpaired two-sample t-test, the present study was powered to detect an anticipated small-to-moderate effect size of 0.4 at 12 weeks with 90% power and probability type I error of 0.05 (two-sided). Patients will be nested within the four intervention sites, which will introduce some within-site correlation that could decrease the efficiency of these estimators. Thus, a variance inflation factor of 20% was incorporated in the sample size estimation. Additionally, it is predicted that this study will have missing data due to patients discontinuing participation in the study and death. Based both on the research team's past experiences conducting studies in this population and already published perioperative mortality data, we assumed a dropout rate of 11% and a mortality rate at 12 weeks of 3%; thus, patient completion rate was estimated to be 0.86. Together, with the assumptions outlined here, the estimated sample size needed for the present study is a total of 186 patients per arm (372 patients total) and our goal was to recruit 380 patients.

12.7 Criteria for future studies

This is a randomized controlled superiority trial that is powered to detect a difference in the primary outcome, quality of life as measured by FACIT-PAL. It is neither a pilot study nor a part of a sequence of trials.

13. REFERENCES

1. Temel JS, Greer JA, Muzikansky A, et al. Early palliative care for patients with metastatic non-small-cell lung cancer. The New England journal of medicine 2010;363:733-42.
2. Bakitas M, Lyons KD, Hegel MT, et al. Effects of a palliative care intervention on clinical outcomes in patients with advanced cancer: the Project ENABLE II randomized controlled trial. Jama 2009;302:741-9.
3. Zimmermann C, Swami N, Krzyzanowska M, et al. Early palliative care for patients with advanced cancer: a cluster-randomised controlled trial. Lancet (London, England) 2014;383:1721-30.
4. Ferrell B, Sun V, Hurria A, et al. Interdisciplinary Palliative Care for Patients With Lung Cancer. J Pain Symptom Manage 2015;50:758-67.
5. El-Jawahri A, Fishman SR, Vanderklish J, et al. Pilot study of a multimodal intervention to enhance sexual function in survivors of hematopoietic stem cell transplantation. Cancer 2018.
6. Lilley EJ, Khan KT, Johnston FM, et al. Palliative Care Interventions for Surgical Patients: A Systematic Review. JAMA surgery 2016;151:172-83.
7. Radwan RW, Evans MD, Davies M, Harris DA, Beynon J. Pelvic exenteration for advanced malignancy in elderly patients. Br J Surg 2016;103:e115-9.
8. Paul S, Altorki N. Outcomes in the management of esophageal cancer. Journal of surgical oncology 2014;110:599-610.
9. Ragulin-Coyne E, Carroll JE, Smith JK, et al. Perioperative mortality after pancreatectomy: A risk score to aid decision-making. Surgery 2012;152:S120-7.
10. Kneuertz PJ, Pitt HA, Bilimoria KY, et al. Risk of morbidity and mortality following hepato-pancreato-biliary surgery. Journal of gastrointestinal surgery : official journal of the Society for Surgery of the Alimentary Tract 2012;16:1727-35.
11. Pezzilli R, Falconi M, Zerbi A, et al. Clinical and patient-reported outcomes after pancreatoduodenectomy for different diseases: a follow-up study. Pancreas 2011;40:938-45.
12. Martin RC, Brown R, Puffer L, et al. Readmission rates after abdominal surgery: the role of surgeon, primary caregiver, home health, and subacute rehab. Annals of surgery 2011;254:591-7.
13. Kozower BD, Sheng S, O'Brien SM, et al. STS database risk models: predictors of mortality and major morbidity for lung cancer resection. The Annals of thoracic surgery 2010;90:875-81; discussion 81-3.
14. Kelley AS, Morrison RS. Palliative Care for the Seriously Ill. The New England journal of medicine 2015;373:747-55.
15. Dying in America: improving quality and honoring individual preferences near the end of life. Military medicine 2015;180:365-7.
16. Elsayem A, Swint K, Fisch MJ, et al. Palliative care inpatient service in a comprehensive cancer center: clinical and financial outcomes. Journal of clinical oncology : official journal of the American Society of Clinical Oncology 2004;22:2008-14.
17. Temel JS, Greer JA, Admane S, et al. Longitudinal perceptions of prognosis and goals of therapy in patients with metastatic non-small-cell lung cancer: results of a randomized study of early palliative care. Journal of clinical oncology : official journal of the American Society of Clinical Oncology 2011;29:2319-26.
18. Sun V, Grant M, Koczywas M, et al. Effectiveness of an interdisciplinary palliative care intervention for family caregivers in lung cancer. Cancer 2015;121:3737-45.
19. Brumley R, Enguidanos S, Jamison P, et al. Increased satisfaction with care and lower

costs: results of a randomized trial of in-home palliative care. Journal of the American Geriatrics Society 2007;55:993-1000.
20. Smith TJ CP, Cassel B, Penberthy L, Hopson A, Hager MA. A high-volume specialist palliative care unit and team may reduce in-hospital end-of-life care costs. Journal of palliative medicine 2003;5:699-705.
21. Greer JA, Pirl WF, Jackson VA, et al. Effect of early palliative care on chemotherapy use and end-of-life care in patients with metastatic non-small-cell lung cancer. Journal of clinical oncology : official journal of the American Society of Clinical Oncology 2012;30:394-400.
22. Bakitas MA, Tosteson TD, Li Z, et al. Early Versus Delayed Initiation of Concurrent Palliative Oncology Care: Patient Outcomes in the ENABLE III Randomized Controlled Trial. Journal of clinical oncology : official journal of the American Society of Clinical Oncology 2015;33:1438-45.
23. Cassell J, Buchman TG, Streat S, Stewart RM. Surgeons, intensivists, and the covenant of care: administrative models and values affecting care at the end of life--Updated. Critical care medicine 2003;31:1551-7; discussion 7-9.
24. Buchman TG, Cassell J, Ray SE, Wax ML. Who should manage the dying patient?: Rescue, shame, and the surgical ICU dilemma. Journal of the American College of Surgeons 2002;194:665-73.
25. Schwarze ML, Bradley CT, Brasel KJ. Surgical "buy-in": the contractual relationship between surgeons and patients that influences decisions regarding life-supporting therapy. Critical care medicine 2010;38:843-8.
26. Pecanac KE, Kehler JM, Brasel KJ, et al. It's big surgery: preoperative expressions of risk, responsibility, and commitment to treatment after high-risk operations. Annals of surgery 2014;259:458-63.
27. King MT, Agar M, Currow DC, Hardy J, Fazekas B, McCaffrey N. Assessing quality of life in palliative care settings: head-to-head comparison of four patient-reported outcome measures (EORTC QLQ-C15-PAL, FACT-Pal, FACT-Pal-14, FACT-G7). Supportive care in cancer : official journal of the Multinational Association of Supportive Care in Cancer 2019.
28. Lyons KD, Bakitas M, Hegel MT, Hanscom B, Hull J, Ahles TA. Reliability and validity of the Functional Assessment of Chronic Illness Therapy-Palliative care (FACIT-Pal) scale. J Pain Symptom Manage 2009;37:23-32.
29. Cella DF, Tulsky DS, Gray G, et al. The Functional Assessment of Cancer Therapy scale: development and validation of the general measure. Journal of clinical oncology : official journal of the American Society of Clinical Oncology 1993;11:570-9.
30. Cella D, Riley W, Stone A, et al. The Patient-Reported Outcomes Measurement Information System (PROMIS) developed and tested its first wave of adult self-reported health outcome item banks: 2005-2008. J Clin Epidemiol 2010;63:1179-94.
31. Cella D, Yount S, Rothrock N, et al. The Patient-Reported Outcomes Measurement Information System (PROMIS): progress of an NIH Roadmap cooperative group during its first two years. Med Care 2007;45:S3-s11.
32. Peterman AH, Fitchett G, Brady MJ, Hernandez L, Cella D. Measuring spiritual well-being in people with cancer: the functional assessment of chronic illness therapy--Spiritual Well-being Scale (FACIT-Sp). Annals of behavioral medicine : a publication of the Society of Behavioral Medicine 2002;24:49-58.
33. Bruera E, Kuehn N, Miller MJ, Selmser P, Macmillan K. The Edmonton Symptom Assessment System (ESAS): a simple method for the assessment of palliative care patients.

Journal of palliative care 1991;7:6-9.
34. Ayanian JZ, Chrischilles EA, Fletcher RH, et al. Understanding cancer treatment and outcomes: the Cancer Care Outcomes Research and Surveillance Consortium. Journal of clinical oncology : official journal of the American Society of Clinical Oncology 2004;22:2992-6.
35. Zarit SH, Reever KE, Bach-Peterson J. Relatives of the impaired elderly: correlates of feelings of burden. The Gerontologist 1980;20:649-55.
36. Sandelowski M. What's in a name? Qualitative description revisited. Research in nursing & health 2010;33:77-84.
37. Sandelowski M. Whatever happened to qualitative description? Research in nursing & health 2000;23:334-40.
38. Cella DF, Bonomi AE, Lloyd SR, Tulsky DS, Kaplan E, Bonomi P. Reliability and validity of the Functional Assessment of Cancer Therapy-Lung (FACT-L) quality of life instrument. Lung Cancer 1995;12:199-220.
