# Supplementary material for: Effect of Perioperative Palliative Care on Health-Related Quality of Life Among Patients Undergoing Surgery for Cancer: A Randomized Clinical Trial
Source: JAMA Netw Open. 2023 May 31;6(5):e2314660. doi: 10.1001/jamanetworkopen.2023.14660 (PMC10233417; doi:10.1001/jamanetworkopen.2023.14660)
Supplement: Supplement 2. — eAppendix. Patient Questionnaire [file jamanetwopen-e2314660-s002.pdf]

Supplementary Online Content

Aslakson RA, Rickerson E, Fahy B, et al. Effect of perioperative palliative care on health-related quality of life among patients undergoing surgery for cancer: a randomized clinical trial. *JAMA Netw Open*. 2023;6(5):e2314660. doi:10.1001/jamanetworkopen.2023.14660

eAppendix. Patient Questionnaire

This supplementary material has been provided by the authors to give readers additional information about their work.

© 2023 Aslakson RA et al. *JAMA Network Open.*

eAppendix. Patient Questionnaire

Clinician PC Visit Summary

Date of visit (MM-DD-YYYY): ____________________

Name of palliative care provider (your name): ____________________

What type of visit was this:

- ☐ In-person visit
  In-person visit – please specify location: ____________________
- ☐ Telephone visit
  Phone call – who participated in the phone call?
  - ☐ Patient
  - ☐ Family member/Friend
  - ☐ Other
    If other person was involved in phone call – please specify: ____________________
- ☐ Other
  Other type of visit – please specify: ____________________

Please indicate which occurred during your visit (check all that apply):

- ☐ Relationship/Rapport building
- ☐ Illness understanding/Education
- ☐ Cancer treatment decisions/Decision making
- ☐ Coping with serious illness
- ☐ Symptom management
- ☐ Advance care planning
- ☐ Other
  Other topic that occurred in your visit (please specify) ____________________

What was the primary (1st) focus of your visit?

- ☐ Relationship/Rapport building
- ☐ Illness understanding/Education
- ☐ Cancer treatment decisions/Decision making
- ☐ Coping with serious illness
- ☐ Symptom management

© 2023 Aslakson RA et al. *JAMA Network Open.*

- ☐ Advance care planning
- ☐ Other

  Other (please specify): ____________________

What was the secondary (2nd) focus of your visit?

- ☐ Relationship/Rapport building
- ☐ Illness understanding/Education
- ☐ Cancer treatment decisions/Decision making
- ☐ Coping with serious illness
- ☐ Symptom management
- ☐ Advance care planning
- ☐ N/A
- ☐ Other

  Other (please specify): ____________________

What was the tertiary (3rd) focus of your visit?

- ☐ Relationship/Rapport building
- ☐ Illness understanding/Education
- ☐ Cancer treatment decisions/Decision making
- ☐ Coping with serious illness
- ☐ Symptom management
- ☐ Advance care planning
- ☐ N/A
- ☐ Other

  Other (please specify): ____________________

Illness understanding/Education – please specify which occurred (check all that apply):

- ☐ Disease understanding
- ☐ Prognosis
- ☐ Review of test or surgery findings
- ☐ Other

  Other (please specify): ____________________

Coping with serious illness – please specify which were discussed (check all that apply):

- ☐ Behavioral (ex. exercise, sleep, hobbies)
- ☐ Spiritual

© 2023 Aslakson RA et al. *JAMA Network Open.*

- ☐ Maintaining/redirecting hope
- ☐ Life review
- ☐ Counseling by palliative care provider
- ☐ Referral for patient to see outside provider (i.e. psychologist, psychiatrist, therapist, etc.)
- ☐ Referral for family member/caregiver to outside provider (i.e. psychologist, psychiatrist, therapist, etc.)
- ☐ Other

  Other (please specify): ____________________

Who was outside referral made to for "coping with serious illness?"

- ☐ N/A – not discussed/no intervention needed
- ☐ Psychiatry referral
- ☐ Psychology referral
- ☐ Social work referral
- ☐ Pastoral counseling referral
- ☐ Other referral

  Other referral (please specify): ____________________

Advance care planning – please specify what occurred (check all that apply):

- ☐ N/A – not discussed/no intervention needed
- ☐ Health care proxy
- ☐ Goals of Care sheet
- ☐ Death planning or discussion
- ☐ Financial planning/discussion
- ☐ Other

  Other (please specify): ____________________

Which symptoms were addressed? (Check all that apply)

- ☐ Pain
- ☐ Dyspnea
- ☐ Depression
- ☐ Anxiety
- ☐ Insomnia
- ☐ Anorexia
- ☐ Fatigue
- ☐ Nausea/Vomiting/Bloating
- ☐ Other

  Other (please specify): ____________________

© 2023 Aslakson RA et al. *JAMA Network Open.*

Pain – How was this symptom addressed? (Check all that apply)

- ☐ N/A – not discussed/no intervention needed
- ☐ Counseling/Patient education
- ☐ Referral
- ☐ Medications
- ☐ Other

  Other (please specify): ____________________

Pain referral – please specify (check all that apply):

- ☐ N/A – not discussed/no intervention needed
- ☐ Interventional pain clinic
- ☐ Physical therapy
- ☐ Integrative therapies (acupuncture, massage, etc.)
- ☐ Psychotherapy (ex. CBT)
- ☐ Other

  Other (please specify): ____________________

Dyspnea – How was this symptom addressed? (Check all that apply)

- ☐ N/A – not discussed/no intervention needed
- ☐ Counseling/Patient education
- ☐ Referral

  Referral (please specify): ____________________
- ☐ Medications
- ☐ Other

  Other (please specify): ____________________

Depression – How was this symptom addressed? (Check all that apply)

- ☐ N/A – not discussed/no intervention needed
- ☐ Counseling/Patient education
- ☐ Referral
- ☐ Medications
- ☐ Other

  Other (please specify): ____________________

Depression referral – made to who? (Check all that apply)

© 2023 Aslakson RA et al. *JAMA Network Open.*

- ☐ N/A – not discussed/no intervention needed
- ☐ Psychiatry/Psychiatric APN or MD
- ☐ Social Work
- ☐ Psychology
- ☐ Chaplaincy
- ☐ Other

  Other (please specify): ____________________

Anxiety – How was this symptom addressed? (Check all that apply)

- ☐ N/A – not discussed/no intervention needed
- ☐ Counseling/Patient education
- ☐ Referral
- ☐ Medications
- ☐ Other

  Other (please specify): ____________________

Anxiety referral – made to who? (Check all that apply)

- ☐ N/A – not discussed/no intervention needed
- ☐ Psychiatry/Psychiatric APN or MD
- ☐ Social Work
- ☐ Psychology
- ☐ Chaplaincy
- ☐ Other

  Other (please specify): ____________________

Insomnia – How was this symptom addressed? (Check all that apply)

- ☐ N/A – not discussed/no intervention needed
- ☐ Counseling/Patient education
- ☐ Referral

  Referral (please specify): ____________________
- ☐ Medications
- ☐ Other

  Other (please specify): ____________________

Fatigue – How was this symptom addressed? (Check all that apply)

- ☐ N/A – not discussed/no intervention needed
- ☐ Counseling/Patient education

© 2023 Aslakson RA et al. *JAMA Network Open.*

- ☐ Referral
  Referral (please specify): ____________________
- ☐ Medications
- ☐ Other
  Other (please specify): ____________________

Nausea/Vomiting/Bloating – How was this symptom addressed? (Check all that apply)

- ☐ N/A – not discussed/no intervention needed
- ☐ Counseling/Patient education
- ☐ Referral
  Referral (please specify): ____________________
- ☐ Medications
- ☐ Other
  Other (please specify): ____________________

Anorexia – How was this symptom addressed? (Check all that apply)

- ☐ N/A – not discussed/no intervention needed
- ☐ Counseling/Patient education
- ☐ Referral
  Referral (please specific): ____________________
- ☐ Medications
- ☐ Other
  Other (please specify): ____________________

Was an additional symptom addressed?

- ☐ Yes
  Please identify other symptom: ____________________
- ☐ No

How was this symptom addressed? (Check all that apply)

- ☐ Counseling/Patient education
- ☐ Referral
  Referral (please specify): ______________________
- ☐ Medications
- ☐ Other
  Other (please specify): ____

© 2023 Aslakson RA et al. *JAMA Network Open.*

Total Time of visit (in minutes): _______________

Did you communicate with the patient's surgeon regarding this visit?

- ☐ Yes
- ☐ No

How did you communicate with the surgeon?

- ☐ Personalized email
- ☐ Phone
- ☐ In person

Compared with the last visit, would you rate the patient's current clinical status as:

- ☐ Improving
- ☐ Stable
- ☐ Declining
- ☐ N/A (this is the first visit with the patient)

How would you assess the patient's ability to tolerate discussions of their expected prognosis?

- ☐ Unable to tolerate
- ☐ Able to tolerate some discussion
- ☐ Able to tolerate extensive discussion
- ☐ Chose not to assess this today
- ☐ N/A – Unable to assess

© 2023 Aslakson RA et al. *JAMA Network Open.*
