# Supplementary material for: Effect of Perioperative Palliative Care on Health-Related Quality of Life Among Patients Undergoing Surgery for Cancer: A Randomized Clinical Trial
Source: JAMA Netw Open. 2023 May 31;6(5):e2314660. doi: 10.1001/jamanetworkopen.2023.14660 (PMC10233417; doi:10.1001/jamanetworkopen.2023.14660)
Supplement: Supplement 3. — Data Sharing Statement [file jamanetwopen-e2314660-s003.pdf]

Data Sharing Statement

Aslakson. Effect of Perioperative Palliative Care on Health-Related Quality of Life Among Patients Undergoing Surgery for Cancer. *JAMA Netw Open*. Published May 31, 2023. doi:10.1001/jamanetworkopen.2023.14660

Data
Data available: Yes
Data types: Deidentified participant data
How to access data: Study data will be stored for potential sharing within the Palliative Care Research Cooperative (PCRC); requests for data sharing can be made directly to the PCRC at their website https://palliativecareresearch.org.
When available: beginning date: 12-31-2023

Supporting Documents
Document types: None

Additional Information
Who can access the data: Researchers may submit requests to the Palliative Care Research Cooperative for use of the data for secondary data analyses
Types of analyses: For secondary data analysis with a specified purpose.
Mechanisms of data availability: The mechanism is to submit a proposal to the Palliative Care Research Cooperative which they review and then use to grant access.
Any additional restrictions: No further restrictions.
